# Experimental study on permeability characteristics of gas-containing raw coal under different stress conditions

**DOI:** 10.1098/rsos.180558

**Published:** 2018-07-04

**Authors:** Dongming Zhang, Yushun Yang, Hao Wang, Xin Bai, Chen Ye, Shujian Li

**Affiliations:** 1 State Key Laboratory of Coal Mine Disaster Dynamics and Control, Chongqing University, Chongqing 400030, People's Republic of China; 2 College of Resources and Environmental Science, Chongqing University, Chongqing 400030, People's Republic of China; 3 College of Mathematics and Statistics, Chongqing University, Chongqing 400030, People's Republic of China

**Keywords:** loading and unloading, gas pressure, post-peak, gas-containing raw coal, permeability characteristic

## Abstract

The present experimental study on permeability characteristics for raw coal under different stress states is implemented by applying the triaxial self-made ‘THM coupled with servo-controlled seepage apparatus for gas-containing coal’; the result indicates that the flow rate of gas in the coal sample gradually decreases with the nonlinear loading of axial pressure and increases with the nonlinear unloading of axial stress and confining pressure. The flow rate, axial stress and confining pressure curves all satisfy the negative exponential function relation. When the sample reaches the peak intensity, the sample will be destroyed and the stress will drop rapidly; then the flow rate of the sample will increase rapidly. At this stage, the flow rate and axial strain show an oblique ‘v' pattern. The flow rate of the coal sample increases nonlinearly with the increase of gas pressure; the relation curve between flow rate and gas pressure satisfies the power function relation. Under the same confining pressure and gas pressure conditions, the larger the axial stress, the smaller the flow rate of the coal sample. Under the same axial stress and gas pressure conditions, the flow rate of the coal sample will first decrease, but then increase as the confining pressure decreases. During the post-peak loading and unloading process, the flow rate of the coal sample will decrease with the loading of confining pressure but increase with the unloading of confining pressure, and there will be an increase in wave shape with the increase in axial strain. The flow rate of each loading and unloading confining pressure is higher than that of the previous loading and unloading confining pressure. At the post-peak stage, the relation curve between the flow rate of the coal sample and the confining pressure satisfies the power function relation in the process of loading and unloading confining pressure.

## 1. Introduction

As an unconventional natural gas attached to the coal seam, coal-bed methane (CBM) has aroused wide public attention. CBM is also called gas, whose main component is methane [1]. CBM is not only a disaster factor and greenhouse gas in coal mining, but also a clean energy [2]. Therefore, the development of CBM will not only effectively reduce the emission of methane and slow down the greenhouse effect, but will also be an effective measure to prevent disasters such as coal and gas outbursts in mines. Currently, the abstraction and collection of gas through construction and drilling is one major method. During the process, gas will be analysed, diffused and transfused. With the constant outburst of gas, the gas pressure under the coal seam will gradually decrease and cause coal seam deformation and permeability changes, thus influencing abstraction and collection efficiency. However, as abstraction and collection goes deeper and deeper, the stress from the land will increase, while gas pressure will decrease, which will cause coal deformation and promote the seepage effect of CBM. It can be concluded that land stress and gas pressure will directly influence the attachment, analysis and seepage process of CBM. Therefore, it is of great significance to systematically study the influence of gas pressure on coal permeability under different stress conditions.

At present, some scholars have conducted research studies on the dynamic evolution of deformation and permeability of coal samples during the development of CBM. In the initial stage of CBM production, the permeability of the coal seam is greatly affected by stress. Huy *et al*. [3] believed that with the increase of effective stress, seepage channels containing microfractures in reservoirs become narrower and some microfractures are even completely closed, which results in a drastic reduction of coal permeability under high effective stress conditions. Laboratory tests show that effective stress leads to an exponential decline in reservoir permeability [4–9]. Thus there is adsorption and expansion of the coal seam under confining pressure and gas pressure, resulting in a significant reduction in permeability [10]. The mechanical behaviour and permeability evolution of the coal sample have been investigated in recent years, and the permeability of the coal seam is affected by a combination of factors such as water content, *in situ* stress, pore pressure and temperature [11–17]. During the mining process, the three-dimensional stress undergoes complex loading and unloading changes, including axial loading and radial unloading [18–20]. Zhang *et al*. [21] investigated the damage evolution and post-peak gas permeability of raw coal under loading and unloading conditions. Jiang *et al*. [22] studied the permeability, acoustic emission characteristics and energy dissipation characteristics of coal under cyclic loading conditions and established damage variables of coal and energy dissipation. Xu *et al*. [23,24] studied the loading and unloading and cyclic loading of coal sample permeability changes. In order to study the response characteristics of coal permeability to pore pressure, Ye *et al*. [25] carried out the seepage experiments under different simulated *in situ* stresses on loading and unloading paths. Ju *et al*. [26] carried out the experimental study on CH_4_ permeability and its dependence on interior fracture networks of fractured coal under different excavation stress paths. Based on the experimental results showing coal sample permeability decreasing with the increase of gas pressure, Wang *et al*. [27], put forward a method for calculating the permeability of coal and rock based on the Klinkenberg effect. Sun *et al*. [28] investigated a fully coupled semi-analytical model for effective gas/water phase permeability during CBM production. To obtain the real physical model characterizing the pore structure of coal rocks and further explore the rules governing the flow of coalbed methane (CBM) using numerical simulation [29]. Wang *et al*. [30] investigated the effect of *in situ* stress and joint on permeability of the coal bed in Linfen Block, China. Qi *et al*. [31] treated coal as transversely isotropic, and developed an anisotropic permeability model by using different directional modulus reduction ratios as the key parameters. Zhu *et al*. [32] extended the dual-permeability model to examine permeability evolution during the injections of different gases. Niu *et al*. [33] studied the coal permeability characteristic by combining laboratory measurements with a simple gas slippage model that explains the rebound phenomenon. Cheng *et al*. [34] believed that tree-shaped borehole fracturing can be used to develop CBM in underground coal mines. Liu *et al*. [35] performed a new inversion approach for obtaining the gas permeability coefficient of coal particles by matching simulation results with experiment data.

Though many researchers have done a lot of research studies on the deformation and permeability of coal samples during loading and unloading, they mostly focus on the influence of *in situ* stress (confining pressure and axial stress) and effective stress on the permeability of coal gas, and there are a few reports about the research on the permeability of raw coal samples considering the change of gas pressure and post-peak loading and unloading confining pressure in the in situ stress field. So, this paper simulates the influence of gas pressure and post-peak loading and unloading confining pressure on the permeability of raw coal by adopting the self-made “THM coupled with servo-controlled seepage apparatus for gas-containing coal”, and the raw coal specimens prepared from 2+3# outburst coal seam of Shamushu coal mine in coal and gas outburst coal mine. The research results can provide certain theoretical guidance for gas draining and outburst prevention for Shamushu coal mine and other similar mines.

## 2. Test apparatus and scheme

### 2.1. Preparation of coal samples

**Figure 1.**
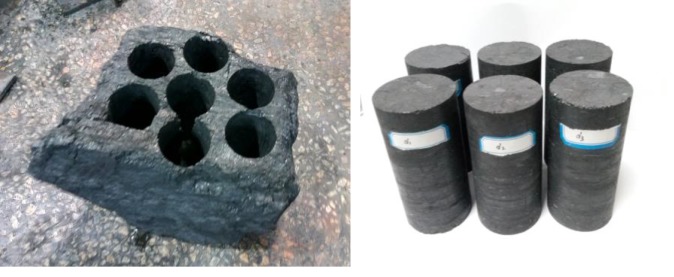
Lump coal and cylindrical raw coal specimens.

The specimens were taken from S3012 working face of the 2 + 3# coal seam of Shamushu coal mine of FuRong company in Yibin, Sichuan province, southwest China. The strike length of the S3012 working face is 752 m, the slope length is 136 m, the depth of the working face is about 450 m, the average dip angle is 4°, the thickness is about 0.8 m ∼ 4.4 m and the average thickness is 3.1 m. The 2 + 3# coal seam experienced a coal and gas outburst at the coal seam, with the coal seam gas content of about 17.37 m^3 ^t^−1^; the methane pressure measured in the field was in the range of 0.8–1.7 MPa, with an average of about 1.32 MPa. The immediate roof is sandy mudstone with a thickness of about 3.0 m. There is a false roof of about 0.4 m between the coal seam and the immediate roof; the false roof is a thin layer of fragile mudstone–argillaceous limestone, which can fall during mining. The main roof consists of sandstone–carbonaceous mudstone with an average thickness of 6 m; the immediate floor is clay-stone, which is soft with water swelling, with an average thickness of 2.8 m; the main floor is sandy mudstone, containing sandstone, with an average thickness of more than 5 m. The industrial indicators of the collected coal are as follows: coal moisture content of 0.96%, dry ash basis of 26.67%, dry ash-free basis of 18.71%, fixed carbon content of 54.28%, gas adsorption constant *a* = 23.9692 m^3^ t^−1^, *b* = 1.2697 m^3^ t^−1^. Large intact coal blocks were drilled from the working face, and they were transported to the laboratory and directly cut into cylindrical specimens 50 mm in diameter and 100 mm long for tests; the lump coal and cylindrical raw coal specimens are shown in figure 1. Vernier callipers were used to measure the specimens' diameter and height, and an electronic balance was used to measure the specimens' mass; the density of coal samples is about 1485.8 kg m^−3^, and the results are given in table 1. The non-parallelism of the two ends of the specimen is less than 0.05 mm, and the machining accuracy meets the requirements of the test methods for the mechanical properties of coal and rocks [36].

**Table 1.** Main parameters of standard cylindrical raw coal specimens.

height (mm)	diameter (mm)	mass (g)
100.24	49.84	292.6
100.02	49.82	291.5
100.12	49.82	290.8
99.98	49.82	288.2
100.10	49.84	288.9
100.04	49.84	288.4

### 2.2. Testing apparatus

**Figure 2.**
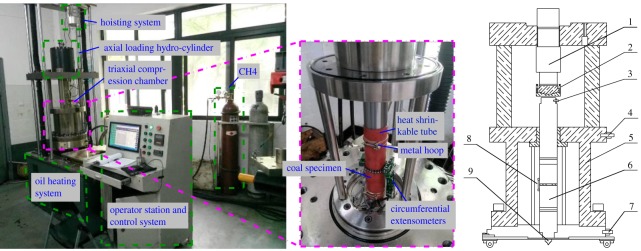
Triaxial stress thermal–hydrological–mechanical coal gas permeameter 1—sensor of axial stress, 2—force plate, 3—gas inlet value, 4—air drain value, 5—pressure vessel, 6—specimen, 7—hydraulic cylinder of axial stress, 8—circumferential extensometers, 9—gas outlet value.

The test was conducted by using a triaxial self-made ‘THM coupled with servo-controlled seepage apparatus for gas-containing coal' [37], as shown in figure 2. This device is composed of the following main components: a servo loading system, pressure chamber, constant temperature oil heating system, gas pressure control system and auxiliary system. The experimental research of the multi-physical coupling of porous media can be performed under different *in situ* stress and mining stress, and temperature and fluid pressure conditions. The stress measurement includes the axial pressure, the confining pressure and the gas pressure, with a maximum axial force of 1000 kN, maximum confining pressure of 60 MPa and maximum gas pressure of 6 MPa. The deformation measurements include axial and radial deformation, with a maximum axial displacement of 60 mm and maximum annular deformation of 12 mm. The gas passing through the specimen is measured with a flow meter. The test temperature is measured with the temperature sensor, and the test temperature ranges from room temperature to 110°C. The accuracy of the measurement system is ±1% for stress, ±1% for deformation and ±1% for temperature control.

### 2.3. Scheme design

**Figure 3.**
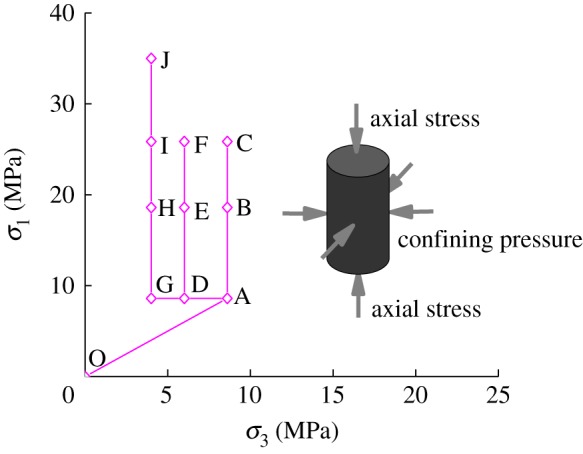
Stress paths in the experiments.

The coal mass is in a state of three-dimensional stress equilibrium before mining, and the three-dimensional stress state of the coal mass undergoes complex changes as the mining progresses, including axial loading and radial unloading. Therefore, the test takes full account of the gas permeability characteristics of the loaded coal under different working conditions. Based on the distribution law of mining stress in the S3012 working face, we investigated the mechanical behaviour of the complete stress-strain process and the permeability of gas-containing raw coal under the combined action of axial stress, confining pressure and gas pressure. The axial stress and confining pressure reflect the change of mining stress in the process of gas drainage, and the change of gas pressure reflects the process of gas desorption, diffusion and seepage in the coal seam. Therefore, the three-way loading and unloading stress path is shown in figure 3, which is described as follows:

(1) An isotropic *in situ* stress state of *σ*_1_ = *σ*_2_(*σ*_3_) = 8.6 MPa is applied to the specimen (this is the loading path OA). Then, the gas pressure is adjusted as p_1_ = 1.0 MPa; to fully absorb the sample for 12 h, the gas outlet value is opened; when the flow rate is stable, the gas pressure is adjusted by the pressure relief valve from 0.5 MPa to 1.0 MPa to 1.5 MPa to 0.5 MPa, respectively. Then, with the maintenance of the confining pressure at 8.6 MPa and the gas pressure at 0.5 MPa, axial stress is loaded phase by phase up to 18.6 MPa (AB stage) and 25.85 MPa (BC stage). Afterwards, the axial stress is unloaded to 8.6 MPa (CA stage). Then the confining pressure is unloaded to 6.0 MPa (AD stage), with the axial stress level of 8.6 MPa.
(2) Maintaining the constant confining pressure at 6.0 MPa and gas pressure at 0.5 MPa, the axial stress is loaded phase by phase to 18.6 MPa (DE stage) and 25.85 MPa (EF stage). Afterwards, when the flow rate is stable, the axial stress is unloaded to 8.6 MPa (FD stage). Then the confining pressure is unloaded down to 4.0 MPa (DG stage), with the axial stress maintained at 8.6 MPa.
(3) Maintaining a constant confining pressure at 4.0 MPa and gas pressure at 0.5 MPa, the axial stress is loaded phase by phase to 18.6 MPa (GH stage) and 25.85 MPa (HI stage).
(4) Maintaining the confining pressure constant at 4.0 MPa and the gas pressure at 0.5 MPa, the axial stress control is changed to displacement control at a speed of 0.1 mm min^−1^, and the axial stress is continuously loaded until the specimen is destroyed (this is the loading path IJ).
(5) After the sample is destroyed, cycle loading and unloading confining pressure tests are carried out four times; meanwhile, the axial stress is loaded at a displacement loading rate of 0.1 mm min^−1^.

After the session AB is completed, its gas pressure was changed with the steps of 0.5 MPa → 1.0 MPa → 1.5 MPa → 0.5 MPa (maintaining each step's gas pressure for 5 min to stable the flow rate).The same test was carried out for all the stages of BC,CA + AD, DE, EF, FD + DG, GH and HI. Particularly, during the process of the above tests, the loading and unloading velocity of axial stress was strictly kept at 0.05 kN s^−1^, the unloading rate of the pre-peak confining pressure at 0.02 MPa s^−1^, the post-peak loading and unloading velocity of the confining pressure at 0.05 MPa s^−1^.

## 3. Experiment result and theoretical analysis

### 3.1. Deformation features during loading and unloading

**Figure 4.**
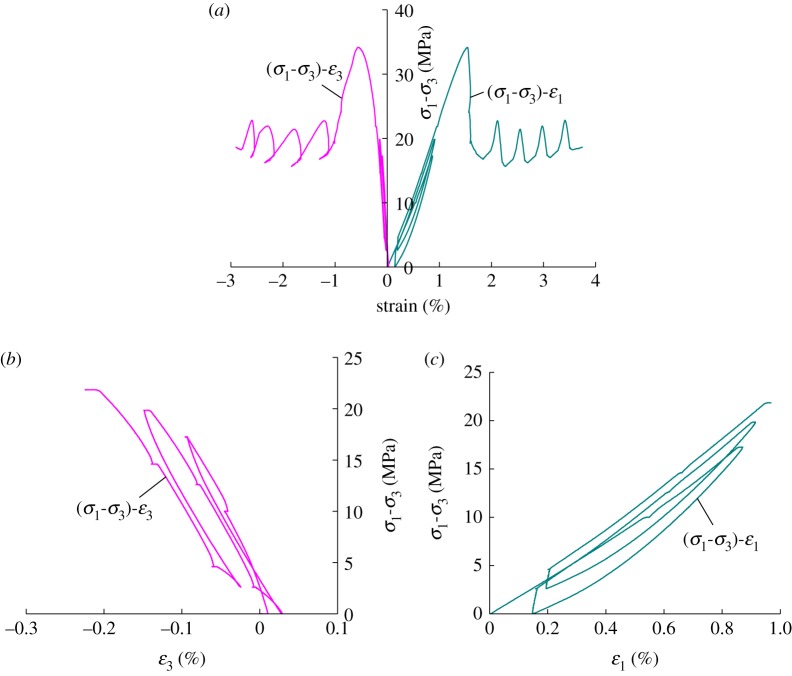
Deviatoric stress–strain curves of coal under loading and unloading conditions. (*a*) Deviatoric stress–strain curves of the coal specimen. (*b*) Deviatoric stress–radial strain curves of the coal sample. (*c*) Deviatoric stress–axial strain curves of the coal specimen.

Figure 4 presents the deviatoric stress–strain curves of coal under the loading and unloading conditions.

(1) The analysis on pre-peak loading and unloading axial stress and unloading confining pressure: it can be seen from figure 4 that the sample exhibits compression deformation in the axial direction while expansion deformation in the radial direction during pre-peak loading axial stress. The sample exhibits rebound in both the axial direction and the radial direction during unloading axial pressure. During unloading confining pressure, the sample exhibits both axial compression and radial expansion. The strains under different stresses are listed according to the experiment results in figure 4, and the details are indicated in table 2.

**Table 2:** Deformation parameters of the coal sample under different stress of pre-peak conditions.

confining pressure (MPa)	axial stress (MPa)	axial strain *ε*_1_ (%)	radial strain *ε*_3_ (%)
8.6	8.6	0	0
	18.6	0.542	0.005
	25.85	0.857	0.094
	8.6	0.148	0.029
6.0	8.6	0.164	0.006
	18.6	0.615	0.077
	25.85	0.906	0.142
	8.6	0.194	0.024
4.0	8.6	0.207	0.055
	18.6	0.656	0.132
	25.85	0.955	0.209
	peak intensity	1.54	0.558

It can be seen from table 2 that after loading and unloading axial stress for the first time, the strain in the axial direction of the sample has increased by 0.148 × 10^−2^ and that in the radial direction has increased by 0.029 × 10^−2^, while after unloading confining pressure the strain in the axial direction has increased by 10.67% and that in the radial direction has decreased by 79.99%. After loading and unloading axial stress for the second time, the strain in the axial direction of the sample has increased by 0.031 × 10^−2^ and that in the radial direction has increased by 0.019 × 10^−2^, while after unloading confining pressure the strain in the axial direction has increased by 6.6% and that in the radial direction has decreased by 126.33%. It can be concluded that under the same axial stress, the lower the confining pressure, the larger the strain on the sample in the axial and radial direction, and the influence of confining pressure on radial strain will be larger than that on axial strain. After loading and unloading axial pressure for the second time, axial strain and radial strain have increased more than the first time. The reason is that during loading and unloading, though the axial stress and strain are within the elastic range, the coal sample is not completely elastically deformed but plastically deformed. The coal sample is compressed by axial strain and its internal gaps get closed, then the strain increases with the increase of axial stress. And during unloading axial pressure, the internal gaps in the coal sample gradually open and the strain will gradually rebound during the unloading of axial stress, but the closed loop curve will not be generated and the curves of the loading and unloading process will not coincide. The internal structure of the coal sample damaged. The confining pressure has a constraining effect on the deformation of coal samples, especially on the transverse deformation. Therefore, during the unloading process of fixed axial pressure against confining pressure, the smaller the confining pressure, the greater the difference of principal strains, the coal sample will be deformed more rapidly and the deformation velocity in the radial direction will be greater than that in the axial direction. When the sample reaches the ultimate strength, the sample will be destroyed and the strain will decrease rapidly. The sample will exhibit apparent expansion in volume, and the axial strain velocity and the radial strain velocity will increase rapidly.

(2) During post-peak loading and unloading confining pressure (4.0 MPa → 9.0 MPa → 4.0 MPa), the axial direction of the sample will be imposed with pressure through displacement, which will cause the axial strain to be linearly increased during the process. The axial direction of the sample will exhibit compression deformation, while during this process, the radial direction of the sample will exhibit compression deformation with the loading of confining pressure and expansion deformation with the unloading of confining pressure. We can learn from table 3 that when loading confining pressure for the first time, the radial strain has decreased by 12.98%, while that for unloading confining pressure has increased by 62.85%. When loading confining pressure for the second time, the radial strain has decreased by 9.40%, while that for unloading confining pressure has increased by 41.311%. When loading confining pressure for the third time, the radial strain has decreased by 4.26%, while that for unloading confining pressure has increased by 20.18%. When loading confining pressure for the fourth time, the radial strain has decreased by 2.49%, while that for unloading confining pressure has increased by 10.04%. It can be concluded that as cyclic loading and unloading times of confining pressure increase, the decrease and increase amount of radial strain after loading and unloading of confining pressure will gradually decrease.

**Table 3.** Strain and flow rate under post-peak loading and unloading confining pressure conditions.

confining pressure (MPa)	axial stress (MPa)	axial strain *ε*_1_ (%)	radial strain *ε*_3_ (%)	flow rate (ml s^−1^)
4	20.86	1.831	−1.294	1.75
9	28.601	2.042	−1.126	0.92
4	19.7	2.263	−1.834	1.92
9	27.37	2.475	−1.661	1
4	20.28	2.688	−2.348	2
9	27.98	2.905	−2.177	1.08
4	21.087	3.124	−2.617	2.17
9	28.9	3.342	−2.551	1.25
4	22.24	3.552	−2.808	2.42

### 3.2. The permeability characteristics of the coal sample during loading and unloading

According to the experimental loading and unloading scheme, the permeability for the coal sample during the loading and unloading process is analysed in two parts: the first part consisting of steps 1, 2, 3 and 4 in the scheme, and the second part, step 5 in the scheme.

#### 3.2.1. Part 1 gas seepage experiment result analysis

**Figure 5.**
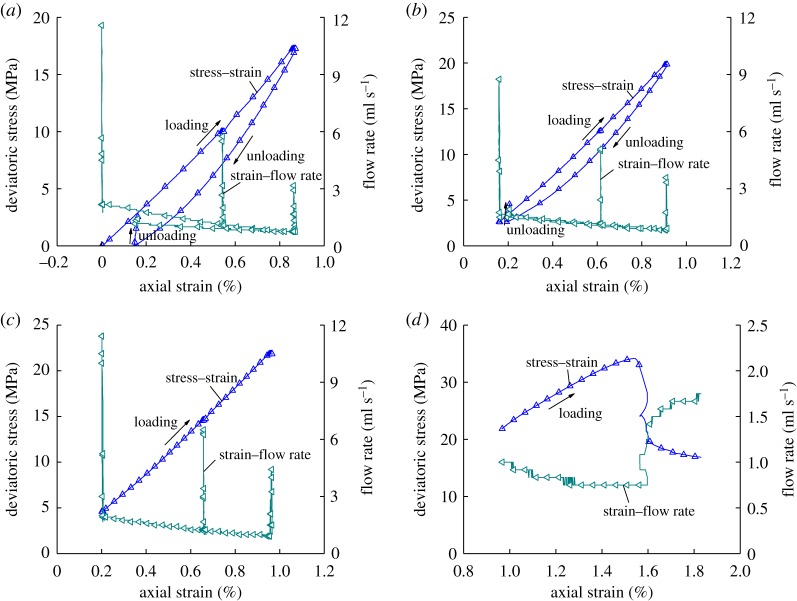
Deviatoric stress–strain–flow rate curves of the first part. (*a*) step 1, (*b*) step 2, (*c*) step 3 and (*d*) step 4.

(1) Analysis of gas seepage characteristics during the loading and unloading process:

Figure 5 shows the first part of stress–strain–flow rate variation curves of coal samples during loading and unloading.

The flow rate under different stresses based on figure 5 was indicated in table 4.

**Table 4.** Flow rate under different stress states.

		gas pressure (MPa)		
confining pressure (MPa)	axial stress (MPa)	0.5	1.0	1.5
8.6	8.6	2.17	5.67	11.58
	18.6	1.08	2.67	5.83
	25.85	0.75	1.67	3.17
	8.6	1.17		
6.0	8.6	1.5	3.92	8.67
	18.6	1.08	2.42	5.17
	25.85	0.83	1.75	3.67
	8.6	1.5		
4.0	8.6	1.92	5.17	11.58
	18.6	1.25	2.92	6.5
	25.85	0.92	2.08	4.5
	peak intensity	0.75		
	residual strength	1.72		

We can learn from table 4 that the flow rate of the coal sample gradually decreases with the loading of axial pressure and gradually increases with the unloading of axial pressure and confining pressure. It also gradually increases as the gas pressure increases. Under the same confining pressure and gas pressure, the larger the axial pressure, the smaller the flow rate of the coal sample. The likely reason is that with the increase in the axial pressure, the coal sample is gradually compacted, its internal gaps decrease, it will become more difficult for gas to pass through the coal sample and hence its flow rate will decrease. During unloading axial pressure and confining pressure, the micro gaps and micro holes inside the coal sample will gradually open with the release of pressure, the internal porosity of the coal sample will increase, it will be less difficult for gas to pass through the coal sample and hence its flow rate will increase. As the gas pressure increases, gas will fill the micro-cracks and hinder the shrinkage inside the coal sample from compression while promoting internal cracks to expand. During the experiment, gas is fully attached to the coal sample. The internal gas pressure of the coal sample can approximately be regarded as a uniform distribution; therefore, as the gas pressure increases, the pressure gradient of gas gradually increases at both ends of the coal sample, more gas will pass through the section of the coal sample per unit time and the corresponding flow rate will be greater. During the process of loading axial pressure through displacement, the cracks inside the coal sample will shrink and the flow rate will gradually decrease. When the coal sample reaches the ultimate strength, it will be destroyed. The strain will decrease rapidly and the crack expansion changes just as in the yielding phase, new cracks will be produced in the axial direction and the flow rate of the sample will increase rapidly. When the sample exhibits residual strength, the changes in the flow rate of the sample will decrease while showing an increasing trend. The flow rate and axial strain change show an oblique ‘v' shape change.

**Figure 6.**
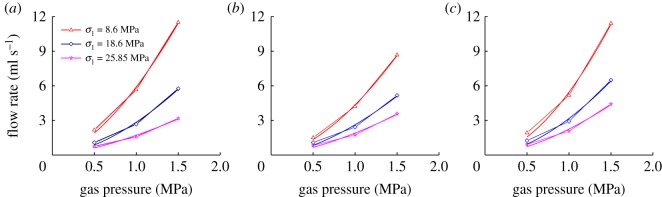
Variation curves of flow rate with gas pressure under different axial stress. (*a*) σ_3_=8.6 MPa, (*b*) σ_3_=6.0 MPa and (*c*) σ_3_=4.0 MPa.

(2) Analysis of the influence of gas pressure on the flow rate of the sample:

According to the results of 1, 2 and 3 in the experimental scheme, the change curves of the flow rate under different axial and confining pressures with gas pressure are shown in figure 6.

We can learn from figure 6 that as the gas pressure increases, the flow rate of the sample will gradually increase. Under the same confining pressure, as the axial pressure increases, the flow rate of the sample will decrease first and then increase. When the confining pressure is unloaded from 8.6 to 6.0 MPa, the sample has experienced the loading and unloading of axial pressure for one time. When the axial pressure is loaded, the internal micro gaps and holes of the sample will be closed, but the unloading axial pressure will not enable compacted holes and cracks to fully restore to the original shape; then the flow rate of the sample will not be restored, which means that the sample is plastically deformed and the internal parts have been damaged. However, during the unloading process of confining pressure, the flow rate of the sample will gradually increase, but it will always be less than that when the confining pressure is 8.6 Mpa. When the confining pressure is unloaded from 6.0 to 4.0 MPa, the sample will experience another period of loading and unloading of axial pressure; now the original cracks inside the sample will be expanded and new micro-cracks will emerge; then the flowing channel of gas will be increased, which means when the confining pressure is unloaded to 4.0 MPa, the flow rate of the sample will increase.

The relation curve between the flow rate of gas and gas pressure is shown in figure 5. The relation between the seepage velocity and gas pressure of raw coal under different axial pressure and confining pressure is presented in table 5.

**Table 5:** Relational expression between flow rate and gas pressure.

confining pressure (MPa)	axial stress (MPa)	relational expression	*R*^2^
8.6	8.6	$v=5.9005p^{1.6297}$	0.9971
	18.6	$v=2.8449p^{1.7117}$	0.9931
	25.85	$v=1.7128p^{1.4823}$	0.9873
6.0	8.6	$v=4.3482p^{1.6904}$	0.9984
	18.6	$v=2.6259p^{1.6387}$	0.9882
	25.85	$v=1.9057p^{1.5181}$	0.9862
4.0	8.6	$v=5.450p^{1.806}$	0.9954
	18.6	$v=3.1666p^{1.7444}$	0.989
	25.85	$v=2.2502p^{1.634}$	0.989

It can be seen from table 5 that under different axial and confining pressures, the flow rate and gas pressure are nonlinearly related. As the gas pressure increases, the flow rate of the coal sample will increase in a power function. When the axial pressure and confining pressure are fixed, the relation between the seepage velocity of the coal sample and the gas pressure can be expressed as follows:

3.1 $$v=\text{a}p^{b},$$

where *v* is seepage velocity, in ml s^−1^; *P* is the gas pressure, in MPa; and a, b are the fitting coefficients.

**Figure 7.**
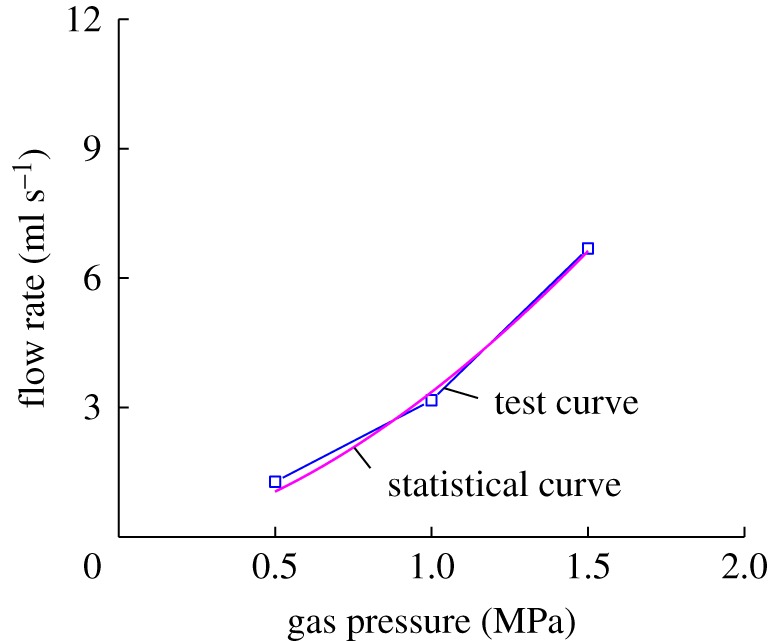
Comparison of empirical formula and test curve of average flow rate and gas pressure.

Based on the average of the flow rates of the coal sample under the same gas pressure, the empirical relation between the flow rate of the coal sample and the gas pressure can be expressed as: $v=3.3561p^{1.6506}$, among which p∈[0.5 MPa, 1.5 MPa]. If we compare the empirical formula with the experimental curve, the curves will be found to be identical, as shown in figure 7.

**Figure 8.**
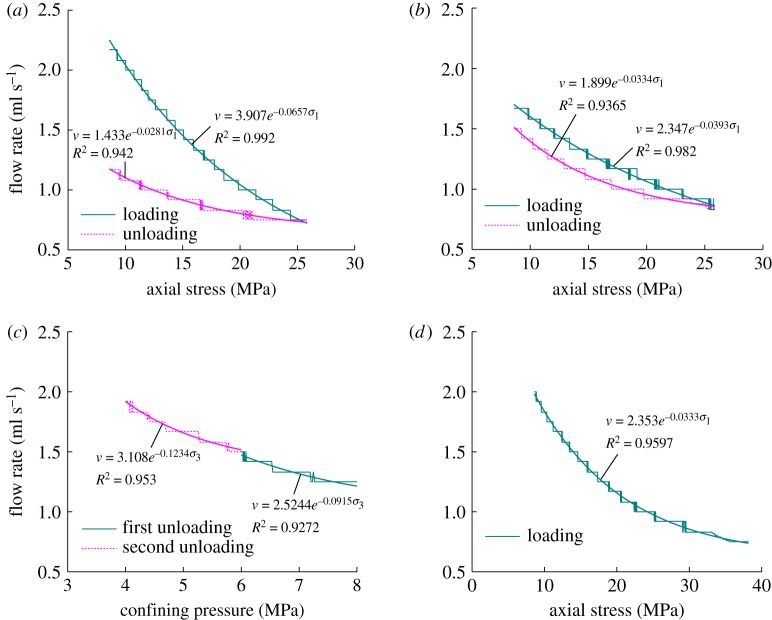
Fitting curves of the flow rate of the sample under loading and unloading conditions. (*a*) First loading and unloading axial stress, (*b*) second loading and unloading axial stress, (*c*) unloading confining pressure twice and (*d*) third loading axial stress.

(3) The flow rate fitting relation curves of the coal sample under *in situ* stress:

If the influence of changes in gas pressure on the flow rate of the coal sample is ignored, the change rules in the flow rate of coal sample under a constant gas pressure of 0.5 MPa and land stress are shown in figure 8. The flow rate of the coal sample will nonlinearly decrease with the loading of axial pressure, but nonlinearly increase with the unloading of axial pressure and confining pressure. We can learn from the fitting relation curve between the flow rate of the coal sample and axial pressure as well as confining pressure that they both satisfy the negative exponential function relation.

(4) Stress sensitivity analysis of coal permeability:

According to the test results in table 3, and the analysis of the test results in §2 and 3, when the gas pressure was increased from 0.5 MPa to 1.0 MPa and 1.5 MPa, respectively, (that is, the gas pressure was adjusted 2.0 times and 3.0 times the initial gas pressure, respectively), the average flow rate of the sample increases to 2.4 and 5.12 times of the initial flow rate. When the axial stress was loaded phase by phase from 8.6 MPa to 18.6 MPa and 25.85 MPa, respectively, (that is, the axial stress was loaded to 2.16 times and 3.0 times of the initial axial stress, respectively), the flow rate of the specimen is reduced to 57.79% and 40.08% of the initial flow rate, respectively. Correspondingly, when the axial stress was unloaded to 0.72 times and 0.33 times of the initial axial stress, respectively, the flow rate increases to 1.46 and 1.963 times of the initial flow rate, respectively. While the confining pressure was unloaded from 8.6 MPa to 6.0 MPa and 4.0 MPa, respectively, (that is, the confining pressure was unloaded to 0.7 times and 0.47 times of the initial confining pressure, respectively), the average flow rate of the specimen becomes 0.92 times and 1.12 times of the initial flow rate, respectively.

In summary, the flow rate of the coal sample gradually decreases with the loading of axial pressure, and gradually increases with the unloading of axial stress and confining pressure. It also gradually increases as the gas pressure increases. Under the same confining pressure and gas pressure, the larger the axial stress, the smaller the flow rate of the coal sample. Under the same confining pressure and axial stress, the larger the gas pressure, the larger will be the flow rate of the coal sample. Under the same axial stress and gas pressure conditions, the flow rate of the coal specimen first decreases and then increases slowly. Therefore, the gas pressure is most sensitive to the flow rate of the coal specimen, followed by the confining pressure and axial stress.

#### 3.2.2. Part 2 gas seepage experiment result analysis

**Figure 9.**
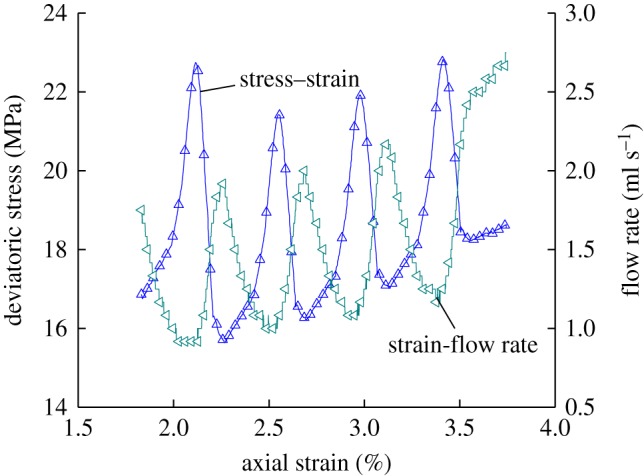
Deviatoric stress–strain–flow rate curves of the second part.

During loading and unloading, the change curve between the deviatoric stress, strain and flow rate of the coal sample in the second part is shown in figure 9.

**Figure 10.**
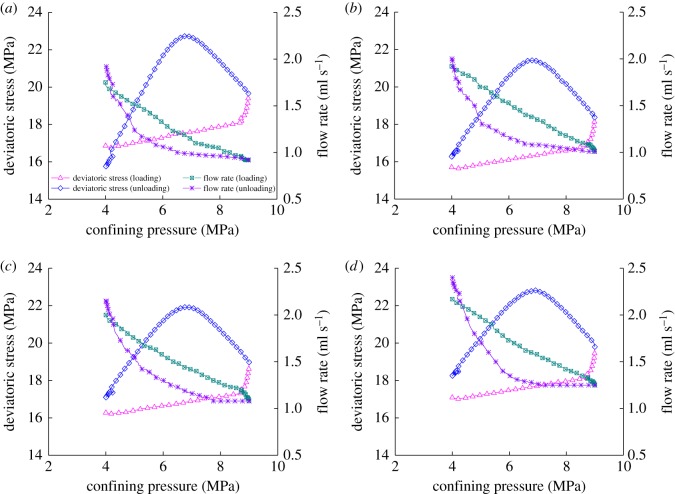
Deviatoric stress–confining pressure–flow rate curves under post-peak loading and unloading confining pressure. (*a*) First loading and unloading confining stress, (*b*) second loading and unloading confining stress, (*c*) third loading and unloading confining stress and (*d*) fourth loading and unloading confining stress.

The flow rate of coal sample under different stresses listed based on figure 9 of the relation curve between stress, strain and flow rate are as presented in table 3. When loading confining pressure for the first time, the flow rate of the sample has decreased by 47.43%, while that for unloading confining pressure has increased by 1.09 times. When loading confining pressure for the second time, the flow rate of the sample has decreased by 47.92%, while that for unloading confining pressure has doubled. When loading confining pressure for the third time, the flow rate of the sample has decreased by 46.0%, while that for unloading confining pressure has increased by 1.1 times. When loading confining pressure for the fourth time, the flow rate of the sample has decreased by 42.4%, while that for unloading confining pressure has increased by 0.94 times. It can be concluded that during the process of post-peak loading and unloading confining pressure, the flow rate of the sample will gradually decrease with the loading of confining pressure, but gradually increases with the unloading of confining pressure. Moreover, the flow rate of the coal sample will increase in wave shape with the increase in the axial strain, which means that the flow rate of each loading and unloading confining pressure is higher than that of the previous loading and unloading confining pressure. The likely reason is that the sample has been destroyed of post-peak loading and unloading confining pressure. Macro-cracks have been produced inside the sample, and axial pressure has been imposed from the axial direction of the sample through displacement. Then, the loading of confining pressure enables macro-cracks to gradually close, causing the flow rate of the sample to nonlinearly decrease, but the unloading of confining pressure enables macro-cracks to gradually expand; the cracks inside the sample will further expand and new cracks are produced with the combined action from axial stress. The ability for gas to pass through the sample will be enhanced, causing the flow rate of each loading and unloading confining pressure to be higher than that of the previous loading and unloading confining pressure. During the loading and unloading process of confining pressure, the flow rate of the coal sample will be closely related to the confining pressure. The relation curve between principal stress difference, confining pressure and flow rate of the coal sample during post-peak loading and unloading of confining pressure is shown in figure 10.

We can see from figure 10 that the deviatoric stress of the sample basically exhibits linear increases with the loading of confining pressure, while the flow rate of the sample will nonlinearly decrease. When the confining pressure is loaded to 98% of the predetermined value, the deviatoric stress of the sample will increase rapidly, and the flow rate of the sample will decrease rapidly. The likely reason is that the loading of confining pressure against the coal sample from another side will enable cracks and partial holes to be closed or deformed, the circulation channel for gas will become smaller and hence the flow rate of the gas will nonlinearly decrease. When the loading of confining pressure for the sample reaches the predetermined value of 9 MPa, the influence of the experimental system on the loading velocity of confining pressure will automatically decrease; while the axial pressure is loading under constant speed through displacement, the axial stress of the sample will increase rapidly, the corresponding deviatoric stress will increase rapidly and hence the flow rate of the sample will decrease rapidly. With the unloading of confining pressure, the deviatoric stress of the sample will continue to increase. When the unloading of confining pressure reaches 6.86 MPa (76.22% of the original unloading value of confining pressure), the *p* deviatoric stress of the sample will be highest, during which the flow rate of the sample will increase slowly with a relatively slight increase in velocity. If the confining pressure continues to unload, the deviatoric stress will first gradually increase before decreasing, during which the flow rate of the sample will gradually increase, and the increase in velocity will become greater and greater. The likely reason is that during the primary stage of unloading confining pressure, the axial pressure of the sample will continue to increase through displacement, the deviatoric stress difference of the sample will continue to increase, the unloading of confining pressure will not enable the closed cracks to effectively open and hence the flow rate of the sample will increase slowly. When the unloading of the confining pressure reaches a certain value, the axial stress of the sample will begin to decrease with the unloading of confining pressure, which means that the deviatoric stress change will experience a turning point during the post-peak unloading process of confining pressure. The closed cracks and gaps inside the sample will gradually open and expand, and new cracks will be produced, the flow rate of the sample will nonlinearly increase and the increase in velocity will be greater and greater. The difficulty for gas to flow in the coal mainly depends on the maturity of seepage channels, such as cracks and effective holes, inside the seepage agents. The maturity of the seepage channels will be beneficial to the circulation of gas in the coal.

**Figure 11.**
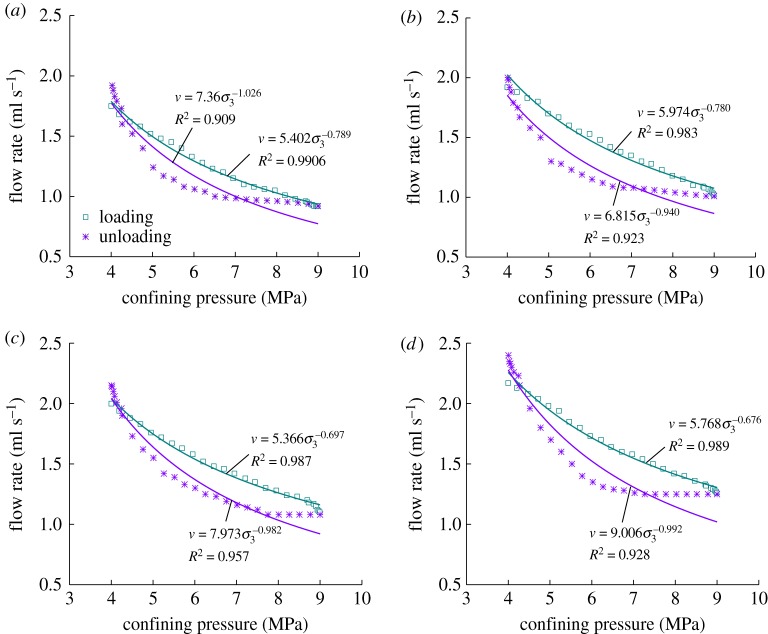
Confining pressure–flow rate fitting curves under post-peak loading and unloading confining pressure. (*a*) First loading and unloading confining stress, (*b*) second loading and unloading confining stress, (*c*) third loading and unloading confining stress and (*d*) fourth loading and unloading confining stress.

The relation curve between the flow rate of the sample and confining pressure during the post-peak loading and unloading of confining pressure is shown in figure 11. The flow rate decreases in power function with the loading of confining pressure but increases in power function with the unloading of confining pressure.

## 4. Conclusion

In the process of pre-peak loading and unloading axial stress and unloading pressure, the loading and unloading curves do not coincide, and the sample exhibits compression deformation in the axial direction but expansion deformation in the radial direction, and the sample exhibits rebound in both the axial direction and the radial direction during unloading axial pressure. During unloading confining pressure, the sample exhibits both axial compression and radial expansion. When the specimen is destroyed, the axial strain and radial strain increase rapidly, and the volume expands significantly. In the process of post-peak loading and unloading confining pressure, the axial strain of the coal sample increases linearly with the increase in the number of times of loading and unloading confining pressure, and the radial strain increases in wave shape.

The flow rate of the coal sample increases nonlinearly with the increase of gas pressure, and the relation curve between gas velocity and gas pressure satisfies the power function relation. Under the same confining pressure and gas pressure conditions, the larger the axial pressure, the smaller the flow rate of the coal sample. Under the same axial pressure and gas pressure conditions, as the confining pressure decreases, the flow rate of coal sample will first decrease, but then increase.

The flow rate of the coal sample gradually decreases with the nonlinear loading of axial pressure and increases with the nonlinear unloading of axial pressure and confining pressure. The flow rate, axial pressure and confining pressure curves all satisfy the negative exponential function relation. When the sample reaches the peak intensity, the sample will be destroyed and the stress will drop rapidly, and hence the flow rate of the sample will increase rapidly. At this stage, the flow rate and axial strain show an oblique ‘v' pattern.

During the post-peak loading and unloading process, the flow rate of the coal sample will decrease with the loading of confining pressure but increase with the unloading of confining pressure. Furthermore, there will be an increase in wave shape with the increase of axial strain. The percolation velocity of each loading and unloading confining pressure is higher than that of the previous loading and unloading confining pressure. After the peak, the relation curve between the flow rate of the coal sample and the confining pressure satisfies the power function relation in the process of loading and unloading confining pressure.

## Data accessibility

We include all the experimental data in the electronic supplementary material, which are available at http://dx.doi.org/10.5061/dryad.bd55173 [38].
